# A Fast Overlapping Community Detection Algorithm with Self-Correcting Ability

**DOI:** 10.1155/2014/738206

**Published:** 2014-03-13

**Authors:** Laizhong Cui, Lei Qin, Nan Lu

**Affiliations:** College of Computer Science and Software Engineering, Shenzhen University, Shenzhen 518060, China

## Abstract

Due to the defects of all kinds of modularity, this paper defines a weighted modularity based on the density and cohesion as the new evaluation measurement. Since the proportion of the overlapping nodes in network is very low, the number of the nodes' repeat visits can be reduced by signing the vertices with the overlapping attributes. In this paper, we propose three test conditions for overlapping nodes and present a fast overlapping community detection algorithm with self-correcting ability, which is decomposed into two processes. Under the control of overlapping properties, the complexity of the algorithm tends to be approximate linear. And we also give a new understanding on membership vector. Moreover, we improve the bridgeness function which evaluates the extent of overlapping nodes. Finally, we conduct the experiments on three networks with well known community structures and the results verify the feasibility and effectiveness of our algorithm.

## 1. Introduction

Community structure is an important field in complex networks research. In the traditional social network, Newman et al. discover the community structure [1–4], which is a group of nodes with dense internal links and sparse connections between groups [3–6]. Later, from the metabolic networks [7] to the large-scale WWW webpage links [8], they are all community structures.

In the exploration of community structure, the crisp division [9, 10] is put forth first by scholars; that is, a node belongs to only one community. In reality, networks are built in different relations, and nodes can be shared by many communities. For example, in human relationship, the relationship of two people may be family, friend, and colleague. When Palla et al. point out the overlap feature of the community [3], a number of soft division algorithms are designed to detect the overlapping community structure, and two main effective means are clique [3, 11–14] and optimization theory [14–16]. The methods based on clique have the high accuracy, but the process is complex. While the optimization algorithms choose the appropriate object function and get a lower complexity. But when and how to finish are ambiguous. Meanwhile, whether a vertex is the overlapping node is not a clear understanding in academic.

In this paper, we propose a fast overlapping community detection algorithm with self-correcting ability through the following contributions. First, we introduce new features of modularity as a new evaluation measurement and explore the advantage of the new weighted modularity in structure through combining the cohesive and density synthetically. Second, we propose three test conditions for overlapping nodes and present a fast overlapping community detection algorithm with self-correcting ability, which consists of two processes. Under the control of overlapping properties, the complexity of our algorithm tends to be approximate linear. Third, we give a new understanding on membership vector to improve the bridgeness function which evaluates the extent of overlapping nodes. To evaluate the feasibility and effectiveness, we implement our approach on three existing networks with the well-known community structures.

The rest of the paper is organized as follows. In Section 2, we describe the details of our new weighted modularity. In Section 3, we propose our fast overlapping community detection algorithm. In Section 4, we explore the estimation to the effect of overlapping node. The experimental results are discussed in Section 5. Finally, Section 6 concludes our work.

## 2. Modularity

### 2.1. The Standard of Overlap

The overlapping node is the vertex that belongs to more than one community. After the analysis on the well-known networks whose structures are also known, we propose three conditions to judge the overlapping node in this paper. In particular, all those are not disrelated. The priority is from the top down, and some nodes will be in accord with several conditions. If a node meets one of the conditions, it shall be an overlapping node.

#### 2.1.1. Addition in Modularity

**Figure 1:**
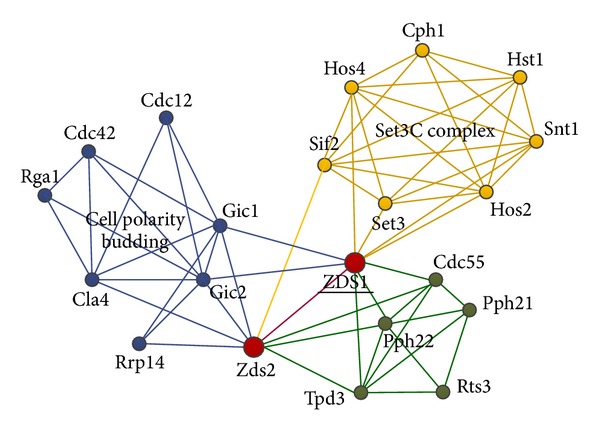
The community structure of Protein reaction network.

**Figure 2:**
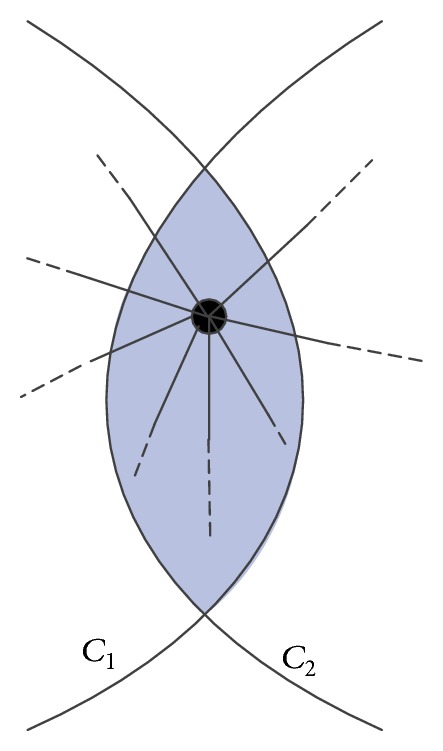
The common overlap.

This is the most common case. Referring to the view of Lázár et al. [17], the node contribution is positive to their communities. The overlapping nodes link many adjacent vertex and belong to less communities (two is general), just as vertex Zds2 shown in Figure 1. In addition, if the adjacent nodes of overlap contain overlapping nodes, they can expand to overlapping region [17] as shown in Figure 2. With the help of this region, all nodes in the sharing area make positive contribution to the belonging communities. The criterion is used in the variation caused by the nodes joining.

#### 2.1.2. Strengthen the Internal Connection

**Figure 3:**
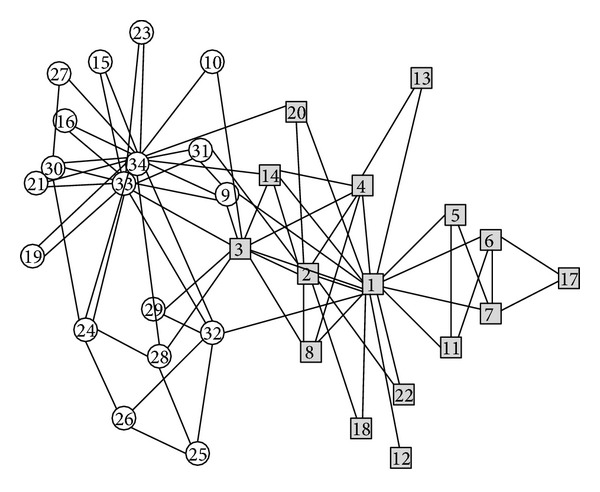
The community structure in Karate club network.

The analysis on the known networks reveals that some core nodes in community may reduce the holistic modularity. Though they have many adjacent nodes, the gain in inner connection is less than the outer connection, which results in the decrease of modularity. However, the internal connection of community is strengthened. In the sparse network, it performs as the addition in density besides the stationarity in modularity whose threshold value is 0.015 in Karate club network as shown in Figure 3. And in the dense network, the proportion of adjacent nodes in community is more than 1/3, according to the research on the Protein reaction network.

#### 2.1.3. The Average Distribution of Belonging Factor

In the networks, the belonging factors of some nodes belong to their communities impartially, which means the adjacent nodes are distributed to the communities averagely. If the degree of node is high, it can be in accord with the previous two cases, just as the Zds2 in Figure 1. Here, the average distribution is not absolute. To be in accord with the membership number, this paper sets a dynamic threshold. Calculating the absolute value of deviation with average distribution, if the sum is less than the dynamic threshold, it is all right.

**Definition 1**

The average distribution of belonging factor should meet the condition in (1), in which *a*_*iC*_*c*__ is the belonging factor of node *i* in community *C*_*c*_ and *n* is the membership number:

(1) $$\begin{array}{c} \overset{n}{\underset{c=1}{\sum }}\left|a_{iC_{c}}-\frac{1}{n}\right|\leq \frac{1}{2n}. \end{array}$$

**Figure 4:**
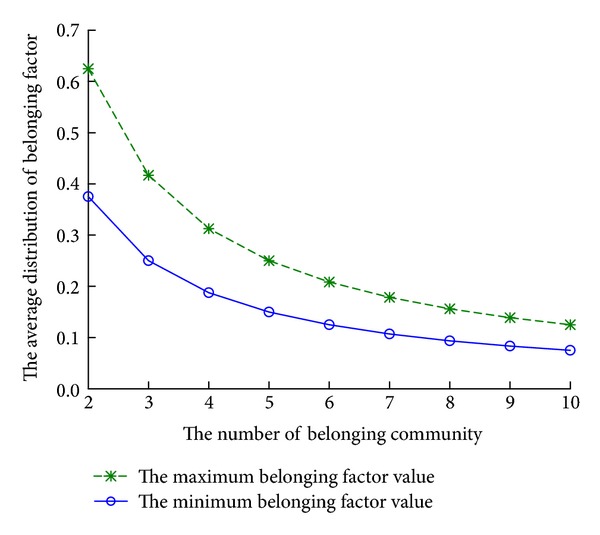
The graph of maximum and minimum value in average distribution.

In (1), 1/*n* is the value of average distribution. With the consideration of the distribution in real network and the accepted empirical value, the dynamic threshold is set as 1/2*n*. With the increasement in membership number, the threshold decreases gradually, and the average feature is more and more obvious in Figure 4.

### 2.2. The New Weighted Modularity

Modularity is the measurement of evaluation and is the preferred object function. Considering various definitions of the past types [18], it summarizes three features after comparing the advantages and disadvantages of each type, which are the fairness, rationality, and independence.

There is the defect in the actual modularity [19], and it disobeys the fairness and rationality. As for the other cases, such as modularity based on node contribution [17] and the overlapping modularity, the weaknesses are discussed [18]. The fitness function [15] handles the inner and outer nodes of community, respectively, defined as (2). For a given community *C*_*i*_, the inner connection is *k*_*C*_*i*__^in^, the outer is *k*_*C*_*i*__^out^, and *α* is the regulatory factor to control the size with the default value 1.0:

(2) $$\begin{array}{c} f_{C_{i}}=\frac{2k_{C_{i}}^{\text{in}}}{{\left(2k_{C_{i}}^{\text{in}}+k_{C_{i}}^{\text{out}}\right)}^{\alpha }}. \end{array}$$

**Figure 5:**
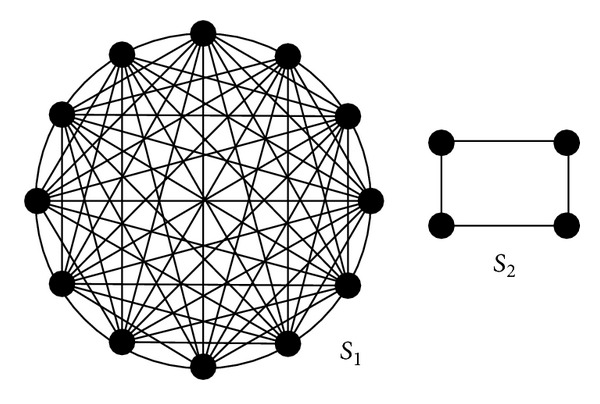
The complete graph *S*_1_ and ring structure *S*_2_.

The fitness function is unfit for modularity, too. That is because it weakens the inner connection which leads to recognize some structures unsuccessfully. For example, the complete graph and the ring structure are shown as Figure 5. They get the same value 1.0, but the structure is quite a bit different. In some subjects like biology or macromolecule, some special structure determines the function.

From the above discussion, in view of network density, we propose a new weighted modularity, which is composed of the density and cohesion.

**Definition 2**

The new weighted modularity is defined as (3), in which the allocation parameter *β* meets 0 < *β* ≤ 0.5 and, for the community *C*_*i*_, *n*_*C*_*i*__ is the number of inner nodes:

(3) $$\begin{array}{c} Q_{C_{i}}=\beta \frac{2k_{C_{i}}^{\text{in}}}{n_{C_{i}}\left(n_{C_{i}}-1\right)}+\left(1-\beta \right)\frac{2k_{C_{i}}^{\text{in}}}{2k_{C_{i}}^{\text{in}}+k_{C_{i}}^{\text{out}}}. \end{array}$$

The modularity of whole network is the average of each community. The research on several typical networks shows that the density of whole network is in a low level which is varying between 0.10 and 0.30. For example, Karate club network [1, 2] is 0.139, Protein reaction network [3] is 0.290, and Dolphins interaction network [2] is 0.084. And here, the rule of parameter allocation *β* obeys Pareto law that is twenty-eighty law. The cohesion gets the 80% weight, and the density gets the 20% weight. With the expansion on the size of networks, the links between nodes are weaker and weaker, and therefore the contribution of density is reduced in modularity.

**Figure 6:**
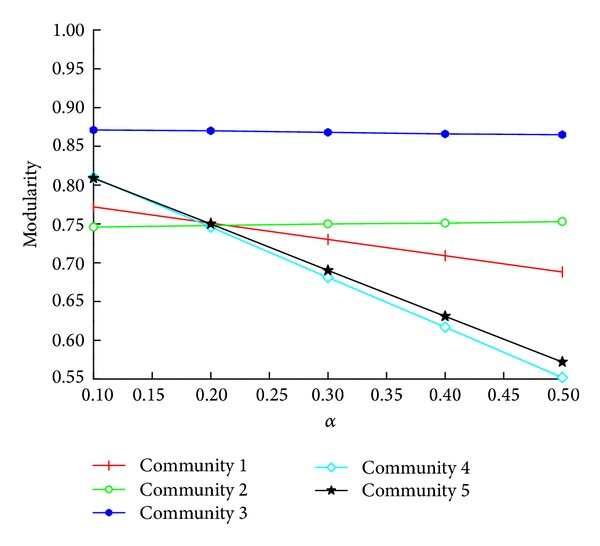
The allocation parameter test graph.

Test on the parameter allocation reveals that when *β* is getting 0.20, the minimum modularity of communities approaches 0.750 in the known networks. However, there is no regular pattern on the other allocation plan and the value is very discrete. As shown in Figure 6, the communities 1–3 are the Protein reaction network, and the communities 4 and 5 are the Karate club network. The detailed communities information of each cluster is shown in Table 1.

Set the allocation parameter *β* as 0.20, and the modularity of communities are as follows.

Through analyzing the test in the known networks, it gets the minimum value of the community structure. In particular, the threshold is for the rough judgment which is not the only condition. In addition, after the node joining, the impacts on the original network, namely the smoothness of the modularity, need to be considered, namely the smoothness of the modularity. The modularity threshold is the last condition in judging, and the detailed introduction is given in next section.

## 3. Fast Overlapping Community Detection Algorithm with Self-Correcting Ability

The traditional community detection methods [3, 11–13, 20] are visiting the nodes repeatedly. But after studying on many overlapping communities structure in some different types, we find out that the vast majority of nodes belong to only one community and the proportion of overlapping nodes is very small. For example, the proportion of overlapping nodes is 3/34 in Karate club network, 2/21 in Protein reaction network, 3/62 in Dolphins interaction network. Visiting the irrelevant nodes again and again results in the reduction of the algorithm efficiency. If we can distinguish the probable overlap nodes or the nonoverlapping nodes, the complexity can be cut down. The algorithm proposed in this paper is based on this idea. It labels the nodes by different property and removes the unrelated nodes to reduce the unnecessary visiting. The fast overlapping community detection algorithm with self-correcting ability is adopted in two stages. The first stage is the initial community discovery, and the second stage is the error detection and correction for specific nodes.

### 3.1. Initial Community Detection Algorithm

#### 3.1.1. Raw Community Detection Algorithm

Referring to the process of local modularity [18, 21], our algorithm selects the root vertex firstly and visits the adjacent nodes in next layer. Then, our algorithm let the eligible nodes join the community and repeats those two steps until there are no qualified nodes. Some studies have verified that a majority of nodes belong to only one cluster in the networks. So, once the node has belonged to the community, it is unnecessary to be visited again. On the basis of this thought, we set each node with two attributes, which are isVisited and isLocated with default value false. They indicate whether a node is visited and located to the community and control the beginning root of the cluster and the range in the next access layer, respectively. Regulating the attributes of nodes, the algorithm proposed in this paper greatly reduces the amount of the adjacent nodes and cuts down the time complexity.

Raw community detection algorithm is composed of the following steps.

1. Pick a node randomly whose isVisited attribute is false as the root of the community, and get the core of the original community.
2. If the count of nodes in the community is greater than 3, install the community model and set the isVisited attribute to be true for all original nodes. Otherwise, set the isVisited of root vertex to be true; then return step 1.
3. Get the adjacent nodes set whose isLocated attributes are false on the basis of parameter nodes which are going to access. And if the count is 0, go next; otherwise, turn to step 5.
4. If the count of nodes in the community is not less than 5, check whether the isLocated attributes are true, and output the original community, and then return to step 1. Otherwise, return to step 1 directly.
5. Access each node in the adjacent nodes set in turn, and set its isVisited attribute value is true.
6. If a node meet the conditions, add it to the current community, and update the community model, and then put it to the next layer to access. Otherwise, return to step 5.
7. If all the nodes are calculated, return to step 3 with the next layer nodes set.

Here, the conditions for a node joining the community are described as follows. Assume that the node joins the community, it meets one of the following conditions: (1) it brings gain in new modularity; (2) it gets addition in density and stable; (3) the rate linked with vertex in community is not less than threshold value (1/3); (4) the modularity is greater than threshold and stable. Theoretical research proves that random selection in nodes has nothing to do with the community structure of network. In other words, every node must belong to a certain community [3]. If root vertex cannot form the community, sign the isVisited attribute to be true, and go on to seek another available root vertex until the core is found.

In this progress, it builds the corresponding community model, which records the detailed information, such as the inner nodes and edges, and the outer edges. If a new node joins the community, it needs to update the community model immediately, which avoids the repetitive computation and lessens the complexity in time.

Moreover, the amount of expansion is not unlimited. From steps 3 to 7, even if there are coincident nodes every time, due to the six-degree theory [8], the diameter of the community is less than 6 and the cycle index is limited, too.

After discovering the community, the algorithm needs to confirm the isLocated attribute. The studies show that the threshold of most nodes' belonging factor in community is 2/3. But in this paper, the threshold of belonging factor is 3/4, and the rate of overlapping edges in all the adjacent edges is less than 1/4. Our algorithm takes a strict criterion to prevent missing the possible overlapping nodes, which is convenient for the error correction algorithm in the next step, just as the number 31 node in Karate club network.

#### 3.1.2. Redistribution Algorithm for Unallocated Nodes

Some studies on the known networks reveal that some nodes have accessed (isVisited = true) in the raw detection stage but failed to be assigned to any detected communities. The reasons are listed as follows: (1) high threshold modularity; (2) close connections among some nodes which form the structure like analogous triangle and any individual vertex cannot meet the requirements. For example, the number 25, number 26, and number 32 nodes are unable to form an independent cluster and need to join the community in union. So, it is necessary to execute the redistribution algorithm for unallocated nodes, which ensures every node belongs to cover.

The way to acquire the unallocated nodes set is calculating the subtraction between the beginning nodes and allocated nodes. For each unallocated node, the flow of the process is described as Algorithm 1.

Owing to the complexity of networks, there may be chain effect. For example, the adjacent nodes connected in sequence just form a chain. To deal with this case, the first node is allocated to the community, and the others are isolated nodes, which will not be participated in the next community detection.

### 3.2. Error Detection and Correction Algorithm for Specific Nodes

The error detection and correction algorithm aims to recognize and check the overlapping nodes, which ensure the accuracy of the result. Here, the specific nodes are those whose isLocated attribute is false. In the process of initial community detection, the timing of nodes joining the community is different, and some core nodes are put in the cluster first. The other will not be identified completely because of missing the information of adjacent nodes. In the following redistribution process, the unallocated nodes decrease the membership value to the community, which may lead to wrongly label to other community. However, the isLocated attribute of wrong classified nodes is false, too. It is the minimum range to execute error correction algorithm on those nodes whose isLocated attribute is false. The researches on some known network show that the more evident the community structure is, the less the specific nodes are. In Karate club network, it is 11/34, in Protein reaction network it is 5/21, and in Dolphins interaction network it is 8/62.

In our algorithm, for every node, the procedure is described as follows.

1. Get the adjacent communities list. If they exist, go next. Otherwise, go to step 5.
2. Select an adjacent community, and verify whether the node meets the conditions to join the community (referring to distribution algorithm), and then decide to join or continue.
3. If all the adjacent communities are tested, then check whether it is equal distribution. If it is, join each adjacent cluster. Otherwise, go next.
4. If the type of joining community is equal distribution, return. Otherwise, continue to go.
5. Get the belonging community set of the node. If the count is 1, return. Otherwise, go next.
6. Choose an unverified community; recalculate the belonging factor which is linked with the community. If it is bigger than the threshold, return.
7. Calculate the modularity variation when removing the node from the cluster. If it is positive, remove the node, return. Otherwise, directly return.

After the initial community detection, the information of nodes membership is completed mostly. The experiments reveal that some overlapping nodes interplay, which would change the node property. That is to say, a new joining node will bring its unallocated adjacent nodes to the same community, and the allocated nodes may be overlapping nodes and expand the overlap region, such as the number 10 and number 3. Therefore, the mutual linking nodes should be extracted, and the error detection and correction algorithm should be executed once again. It could clear up the possible wrong division, which makes the partition reasonable and steady.

Moreover, since the error detection and correction algorithm aims to the specific nodes, when expanding the nodes to the whole network, it is able to detect the validity of other community detection algorithms. If there is no change in node membership, they are just steady and accurate.

## 4. Estimation to the Effect of Overlapping Node

### 4.1. Original Bridgeness

Community modularity is independent with the partition pattern, whether it is hard or crisp. But for the overlapping and nonoverlapping nodes, the roles are different in the network. It needs bridgeness [9] function to evaluate the position and importance of the overlap in the network, and the membership distribution is a major factor. The previous studies suggested that the sum of membership factor is 1.0 [17, 22]. However, there are limitations with this suggestion. In the overlapping communities, the connections among overlaps form the overlapping edges and are the member of multiple communities. So it is calculated more than once and the sum of node membership can be greater than 1.0. For example, the membership vector of Zds1 in Protein reaction network is [0.3, 0.4, 0.4] and the sum of them is 1.1.

**Definition 3**

The sum of node membership vertex meets the condition in (4), in which we assume that the number of belonging communities is *c*:

(4) $$\begin{array}{c} \overset{C_{c}}{\underset{i=1}{\sum }}a_{iC_{c}}\geq 1.0. \end{array}$$

For each node, bridgeness means the degree of sharing in different communities. Nepusz et al. define that it is 0 when the node belongs to only one cluster. And it is 1.0 when sharing equally by the belonging communities. On the basis of membership vertex [*a*_*i*1_, *a*_*i*2_,…, *a*_*ic*_] of overlapping node *i*, after setting the uniform distribution [1/*c*, 1/*c*,…, 1/*c*] as reference vertex, they define the bridgeness [9] as

(5) $$\begin{array}{c} b_{i}=1-\sqrt{\frac{c}{c-1}\overset{c}{\underset{r=1}{\sum }}{\left(a_{iC_{r}}-\frac{1}{c}\right)}^{2}}. \end{array}$$

### 4.2. Improved Bridgeness

The distribution of belonging factors determines the status in network. The more approximate to the uniform distribution it is, the greater the effect is. However, the membership number is a positive and significant element. In addition, the degree of node itself is not an ignorable element. Obviously, Nepusz et al. overlook its own factors. If two nodes all conform to uniform distribution, the bridgeness is 1.0. So it cannot indicate the importance. Moreover, the node contribution of overlap can be positive in more than one community, and 1/*c* fails to express the actual average value of the uniform distribution. So, we set the average value as all the belonging factors. Considering the membership number is lesser than the degree of node, we introduce the improved bridgeness as follows synthetically.

**Definition 4**

Improved bridgeness is defined as follows, in which *k*_*i*_ is the degree of node *i*, and ${\overset{-}{a}}_{i}$ is the value of the actual uniform distribution:

(6) $$\begin{array}{c} b_{i}=1-\sqrt{\frac{c}{c-1}\overset{c}{\underset{r=1}{\sum }}\left(a_{iC_{r}}-{\overset{-}{a}}_{i}^{2}+\frac{1}{c^{2}}+\frac{1}{k_{i}}\right)}. \end{array}$$

As shown in (6), the greater the membership number is, the higher the degree is, and the more similar to uniform distribution the membership vertex is. The sum of variance is less, and then the value of bridgeness is greater, which signifies the important standing in network. The detailed results of comparison and analysis are demonstrated in the next experiments.

## 5. Experiment Results and Analysis

In this section, we evaluate our algorithm with the community structures of three well-known networks, which, respectively, are Karate club network, Protein reaction network, and Dolphins interaction network. Karate club network [1, 15] is the community structure network initially found by Newman, which represents the traditional social research network. Protein reaction network [3] is a network composed of protein metabolism, on behalf of the emerging biology research network, and Palla et al. find the overlapping characteristics of the community through it. Dolphins interaction network [15] is a network built according to interaction information of waters bottlenose dolphin living in New Zealand, which belongs to natural science research field, and many scholars take it as a research subject.

### 5.1. Karate Club Network

Karate club network is the classic interpersonal relationship network, in which 34 members constitute 78 connections. Since the disagreements between members lead to divide into two pies, the network is divided into two distinct communities. In traditional hard classification model, the node can only belong to one community. However, after utilizing our algorithm on this network, we found that three nodes meet the criteria of overlapping node. Since overlap is an important characteristic of complex networks, through analyzing the network structure, the overlap model is more in accord with the actual situation.

During our algorithm implementation process, firstly, it chooses the root node of the community, which is “1,” after three extensions, no subsequent nodes can join in, the original community is found; then it selects the root node, which is the “15”; it ends after three times external extension and completes the found of another community; the obvious overlap nodes {“9”, “31”} are identified. Original community discovery process is described as follows:

 Com1: “1” → “11”, “14”, “22”, “20”, “13”, “18”, “8”, “9”, “2”, “3”, “4”, “5”, “6”, “7” → “12”, “17”, “31”.
 Com2: “15” → “33”, “34” → “16”, “19”, “21”, “23”, “24”, “28”, “30”, “31”, “9” → “27”.

After forced distribution, the set of the unallocated nodes is {“32”, “25”, “26”, “29”, “10”} and none of overlapping nodes is classified by mistake. After the initial community distribution, 29 nodes determine the belonging community.

In the error detection and correction algorithm, the set of the detected nodes is {“34”, “3”, “32”, “9”, “31”, “28”, “29”, “26”, “26”, “25”, “20”, “10”}. Since the adjacency node has not been fully allocated in the process of the initial distribution, most of the nodes lack of adjacency information reference and are unable to determine isLocated = true. In view of the connected closely node {“3”, “9”, “10”}, the theoretical analysis has pointed out this problem that wrong classification may result in the change of the node properties. So, the error detection and correction algorithm should be performed again for such a node, which can eliminate the unreasonable factors and make the classification tend to be stable.

**Figure 7:**
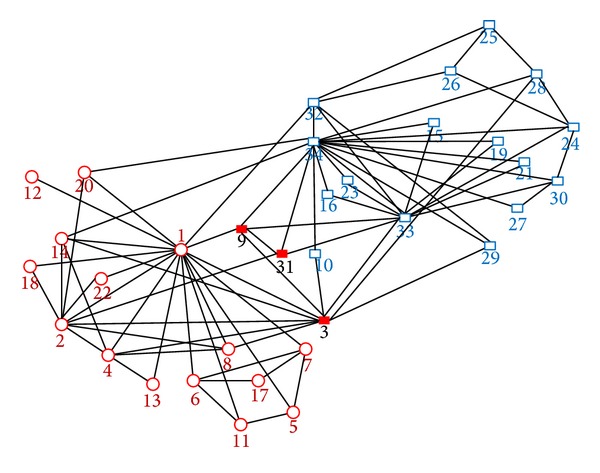
The community structure found by our algorithm in Protein reaction network.

Figure 7 is the final community structure found by our algorithm, which is in accordance with the standard distribution and includes the red solid nodes {“3”, “9”, “31”}, namely, the overlapping nodes. More information of overlapping nodes is shown in Table 2. Compared with number 9 and number 31, both of them belong to two communities. The variance of the former is less than number 31, and the degree is also greater. However, the original bridgeness of number 9 is smaller, which is irrational. And the improved bridgeness displays the difference in nodes, in accord with their own status in the network.

As seen from the membership value, in Karate club network, the sum of overlap node membership degree is greater than 1.0, and each node can increase modularity for belonging community. In addition, the cumulative difference of the absolute value of the node 3 and node 31 membership vertex value and average distribution is less than or equal to a quarter, which are also in accord with the condition of average distribution. Therefore, it also verifies the principle and sequence of overlapping node determination conditions.

### 5.2. Protein Reaction Network

Protein network is built according to metabolism response relationship between the biological protein, containing 21 nodes and 61 sides. It is a typical overlapping community network, the community structure of which is obvious. Through running our algorithm for finding original community, all of the communities are identified, and the nodes are all classified correctly, including high overlapping nodes. The redistribution algorithm needs not to run; thus our algorithm quickly found the overlapping community, which validates the efficiency of our algorithm.

Let us focus on the implementation process of our algorithm. The root node of each community and its subsequent extension process are described as follows:

 Com1: “cdc12” → “gic2”, “cla4”, “gic1” → “cdc42”, “rga1”, “rrp14”, “zds2” → “zds1”.
 Com2: “cph1” → “snt1”, “sif2”, “hst1”, “hos2”, “hos4” → “set3”, “zds1”.
 Com3: “pph21” → “pph22”, “tpd3”, “rts3”, “cdc55” → “zds1”, “zds2”.

**Figure 8:**
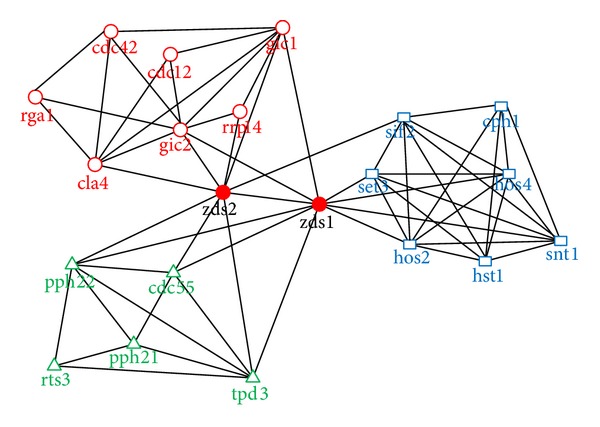
The community structure found by our algorithm in Protein reaction network.

Figure 8 illustrates the three communities found by our algorithm, which is labeled by different shapes and colors. The structure is the same as the standard classification, and the solid red nodes {“zds1”, “zds2”} are the overlapping nodes. The detailed information is shown in Table 3. In particular, they are in accord with the three conditions of overlap, which verifies the effectiveness of our proposed conditions again.

Protein reaction network also demonstrates the irrationality of the original bridgeness. Zds1 node degree and community membership number are all greater than Zds2. But the original bridgeness is still lower than Zds2, and the difference of numerical value is very small, while our improved bridgeness avoids this defect, which is more in line with the node's position in the network community.

### 5.3. Dolphins Interaction Network

**Figure 9:**
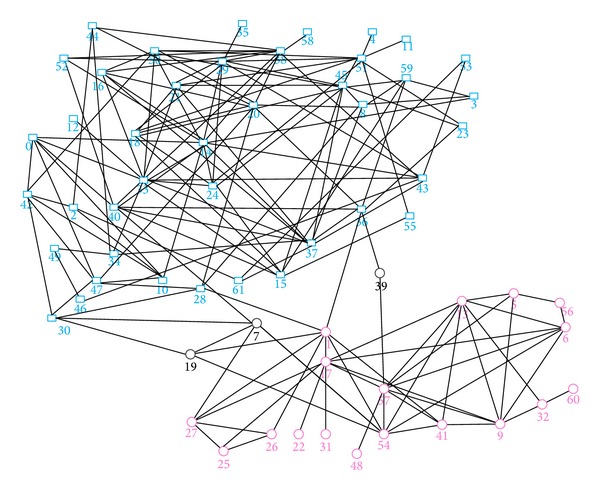
The community structure found by our algorithm in Dolphins interaction network.

Dolphins interaction network is established according to the dolphin interaction information. Its community structure found by our algorithm is shown as Figure 9, in accord with the actual situation. The difference in number of nodes in communities is big, which is 44 and 21, respectively. Through running the algorithm, we found that from the start of the root node “0,” the network cycle needs to extend 7 times to find the large community, and it seems not to be in accord with the small world network model. Through deep analysis, the reason is that some nodes link more closely in the network. If there are no adjacent nodes to join in, the adjacent information is missing and nodes are delayed to join the community, which cause that they are repeatedly visited. This is not in conflict with the six degrees theory in network, because it does not mean extending the network to the seventh layer from the root node. The detailed extension process of raw community is described as follows:

 Com1: 0→ “14”, “47”, “42”, “15”, “10”, “40”→ “2”, “24”, “30”, “52”, “55”, “7”→ “19”, “28”, “29”→ “18”, “20”, “35”, “45”, “51”, “8”→ “11”, “21”, “23”, “3”, “36”, “37”, “4”, “44”, “50”, “59”→ “16”, “33”, “34”, “38”, “39”, “43”, “61”→ “12”, “46”, “49”, “53”, “58”.
 Com2: 13→ “5”, “6”, “41”, “32”, “17”, “57”, “9”, “54”→ “1”, “56”, “60”→ “19”.

Between the starting node 0 in the community Com1 and the last joined node 58 in the community Com1, the shortest distance among them is only 3, in line with the feature of the small world network. Unallocated node set {“27”, “25”, “26”, “22”, “31”, “48”} is allocated correctly, and the specific nodes {“17”, “1”, “27”, “7”, “19”, “25”, “26”, “39”} are achieved stability after completing the error detection and correction algorithm.

The detailed information of overlapping nodes is shown in Table 4. The set of found overlap nodes is {“19”, “39”, “7”}, in which {“19”, “7”} are in accord with multiple overlap conditions. In addition, node 39 conforms to standard uniform distribution, fairly connecting the two communities. Through the comparison of the original and improved bridgeness, since the original bridgeness only considers the membership values, it is unreasonable distinctly, which is illustrated by node 39.

### 5.4. Results Analysis and Discussion

#### 5.4.1. Multiple Constraints of the Algorithm

The other community found that optimization algorithms take the maximize objective function value as the end condition. The initial community detection algorithm proposed in this paper is different and based on extracting the overlapping node features. Multiple conditions and multiple thresholds are constrained to find natural communities, which contains situations as follows: (1) the basic modularity increases; (2) density increases with stability; (3) the connection is greater than the community size threshold; (4) the modularity is greater than the specified threshold with stability; (5) the belonging factors are in accord with uniform distribution. These conditions form an access priority according to the judgement order, and high computational complexity would inspect in the final. Through the reasonable arrangement of the condition priority, it avoids the unnecessary calculation and reduces the complexity.

#### 5.4.2. Operating Efficiency of the Algorithm

In the process of our algorithm running, through controlling the attribute of isLocated, it gradually shrinks the expansion space of the adjacent available nodes and cuts down the repeated access of the nodes, which improve the operation efficiency of our algorithm. For nonoverlapping nodes, theoretical visit is only one time. For the possible overlapping nodes, it only needs to visit and verify the relevant adjacent communities. The attributes are to classify nodes, and it can avoid the repeated visit and compute for the irrespective node effectively.

#### 5.4.3. Strategy of the Algorithm

In the process of discovering natural communities, by establishing community model and updating community information in real time, it avoids the repeated compute community information when the nodes join the community. Most of the time is spent in the process of finding natural community. The subsequent error detection and correction algorithm is a supplement. Since the involved node is less, the complexity of the calculation is lower than community discovery process. So the overall complexity is approximate linear. It shows the node proportion information in each stage during our algorithm running in Table 5.

**Figure 10:**
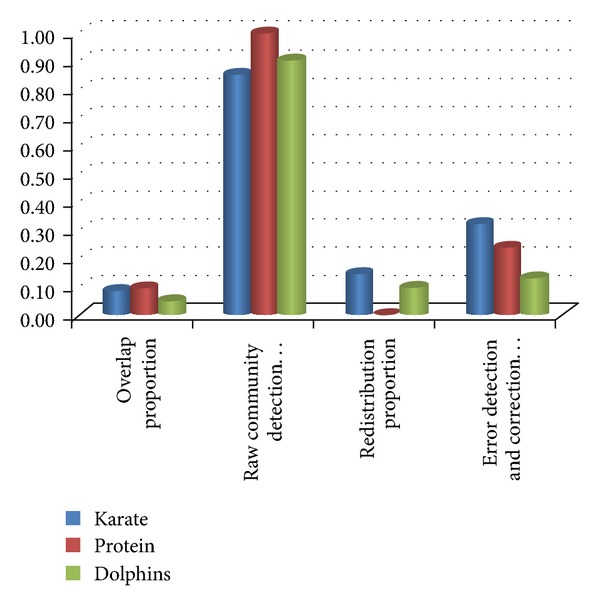
The proportion of the various types of nodes in networks.

In each period of our whole algorithm, the core is the raw community detection algorithm, which detects the main areas of communities. It determines the number of network partition, and affects the efficiency of the algorithm. Compared with the data in the network, the vast majority of nodes in the community are nonoverlap. The overlap nodes and nonoverlap nodes can be identified by the attribute of isLocated, which greatly reduces the repeated visit and calculation to the irrelevant nodes, and the algorithm enables to detect communities rapidly. In Figure 10, more than 80% nodes have been confirmed in the stage of raw community detection. In addition, the more obvious the community structure in the network is, the smaller the proportion of nodes need to redistribute is. Since the number of nodes in error detection and correction stage is limited and the community structure has been clear, it only needs to verify the specific nodes, which do not access large-scale adjacent nodes. So the error detection and correction algorithm has less effect on the increase with overall complexity.

## 6. Conclusions

In this paper, we first put forward the new features of modularity and also show the advantage of the new weighted modularity in structure, which is based on cohesive and density synthetically. The experiments are conducted on the classical networks with well-known community structure, which explore the distribution of the parameter factor. In addition, according to the proportion of overlap in the community, we present a fast overlapping community algorithm with self-correction by setting the nodes with the attributes of isVisited and isLocated, which consists of two stages: (1) the initial community detection algorithm and (2) the error detection and correction algorithm. We also propose an improved bridgeness function to evaluate the extent of overlapping nodes. The experimental results demonstrate that our algorithm is good for the expansion of discovery algorithm when extracting the overlap features. Although our algorithm is already effective, but the later work can be expanded in more different types of networks, to test out appropriate parameter and conclude parameter distribution principle. In addition, the threshold setting in the overlapping node conditions, such as in the modularity, stationarity, and the close connection, is strict in algorithm, which expands the scope of nodes in error detection and correction slightly. However, finding and extracting the new features of overlapping node are the directions in the next step. Through the experiments on the existing network, our algorithm can be applied to large-scale networks in the future.

## Figures and Tables

**Algorithm 1:**
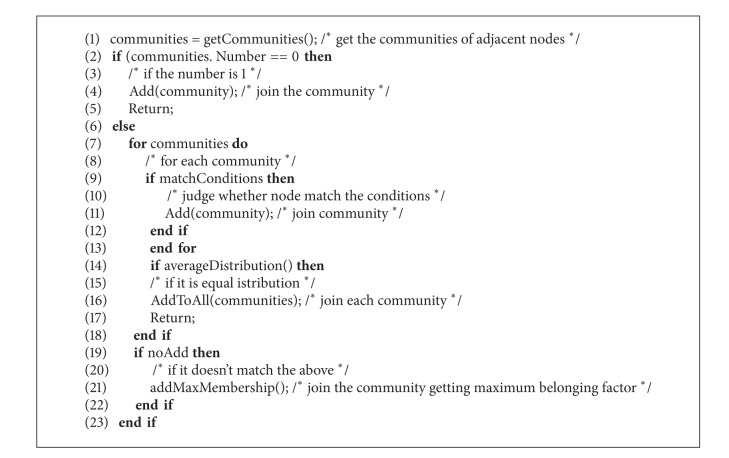
The redistribution algorithm for unallocated nodes.

**Table 1:** The detailed communities information in networks.

Network	Community	Node	Inner link	Outer link	Modularity
Protein reaction network	Community 1	9	21	11	0.751
	Community 2	7	16	11	0.748
	Community 3	8	24	7	0.870

Karate club network	Community 4	18	35	10	0.746
	Community 5	16	33	10	0.750

**Table 2:** The detailed information of overlapping nodes in Karate club network.

Overlapping node	Degree	Membership number	Belonging factor	Original bridgeness	Improved bridgeness
3	10	2	0.60	0.86	0.40
			0.50		

9	5	2	0.60	0.55	0.30
			0.80		

31	4	2	0.50	0.65	0.25
			0.75		

**Table 3:** The detailed information of overlapping nodes in Protein reaction network.

Overlapping node	Degree	Membership number	Belonging factor	Original bridgeness	Improved bridgeness
Zds2	9	2	0.56	0.89	0.39
			0.44		

Zds1	10	2	0.30	0.88	0.53
			0.40		
			0.40		

**Table 4:** The detailed information of overlapping nodes in Dolphins interaction network.

Overlapping node	Degree	Membership number	Belonging factor	Original bridgeness	Improved bridgeness
19	4	2	0.50	0.65	0.25
			0.75		

39	2	2	0.50	1.00	0.14
			0.50		

7	5	2	0.60	0.80	0.33
			0.60		

**Table 5:** The proportion of overlapping nodes and nodes of each stage in the networks.

Community structure	Overlapping proportion	Raw community detection proportion	Redistribution proportion	Error detection and correction proportion
Karate	0.09	0.85	0.15	0.32
Protein	0.10	1.00	0.00	0.24
Dolphins	0.05	0.9	0.10	0.13
